# Correction: Wood-Inhabiting Beetles in Low Stumps, High Stumps and Logs on Boreal Clear-Cuts: Implications for Dead Wood Management

**DOI:** 10.1371/journal.pone.0127220

**Published:** 2015-04-24

**Authors:** The PLOS ONE Staff

## Notice of Republication

This article was republished on April 15, 2015, to correct errors in tables and the body of the article that were introduced during the typesetting process. The publisher apologizes for the errors. Please download this article again to view the correct version. The originally published, uncorrected article and the republished, corrected article are provided here for reference.

## Supporting Information

S1 File Originally published, uncorrected article. (PDF)

S2 File Republished corrected article. (PDF)
