# Supplementary material for: Correction: Wood-Inhabiting Beetles in Low Stumps, High Stumps and Logs on Boreal Clear-Cuts: Implications for Dead Wood Management
Source: PLoS One. 2015 Apr 24;10(4):e0127220. doi: 10.1371/journal.pone.0127220 (PMC4409399; doi:10.1371/journal.pone.0127220)
Supplement: S2 File — (PDF) [file pone.0127220.s002.pdf]

RESEARCH ARTICLE

Wood-Inhabiting Beetles in Low Stumps, High Stumps and Logs on Boreal Clear-Cuts: Implications for Dead Wood Management

Jon Andersson[1]*, **Joakim Hjältén**[1], **Mats Dynesius**[2]

1 Department of Wildlife, Fish and Environmental Studies, Swedish University of Agricultural Sciences, Umeå, Sweden, **2** Department of Ecology and Environmental Science, Umeå University, Umeå, Sweden

* Jon.Andersson@slu.se

OPEN ACCESS

Citation: Andersson J, Hjältén J, Dynesius M (2015) Wood-Inhabiting Beetles in Low Stumps, High Stumps and Logs on Boreal Clear-Cuts: Implications for Dead Wood Management. PLoS ONE 10(3): e0118896. doi:10.1371/journal.pone.0118896

Academic Editor: Danilo Russo, Università degli Studi di Napoli Federico II, ITALY

Received: February 19, 2014

Accepted: January 18, 2015

Published: March 10, 2015

Copyright: © 2015 Andersson et al. This is an open access article distributed under the terms of the Creative Commons Attribution License, which permits unrestricted use, distribution, and reproduction in any medium, provided the original author and source are credited.

Funding: The study was financed by The Swedish Energy Agency (http://www.energimyndigheten.se, Grant to JH). The funders had no role in study design, data collection and analysis, decision to publish, or preparation of the manuscript.

Competing Interests: The authors have declared that no competing interests exist.

Abstract

The increasing demand for biofuels from logging residues require serious attention on the importance of dead wood substrates on clear-cuts for the many forestry-intolerant saproxylic (wood-inhabiting) species. In particular, the emerging harvest of low stumps motivates further study of these substrates. On ten clear-cuts we compared the species richness, abundance and species composition of saproxylic beetles hatching from four to nine year old low stumps, high stumps and logs of Norway spruce. By using emergence traps we collected a total of 2,670 saproxylic beetles among 195 species during the summers of 2006, 2007 and 2009. We found that the species assemblages differed significantly between high stumps and logs all three years. The species assemblages of low stumps, on the other hand, were intermediate to those found in logs and high stumps. There were also significant difference in species richness between the three examined years, and we found significant effect of substrate type on richness of predators and fungivores. As shown in previous studies of low stumps on clear-cuts they can sustain large numbers of different saproxylic beetles, including red-listed species. Our study does, in addition to this fact, highlight a possible problem in creating just one type of substrate as a tool for conservation in forestry. Species assemblages in high stumps did not differ significantly from those found in low stumps. Instead logs, which constitute a scarcer substrate type on clear-cuts, provided habitat for a more distinct assemblage of saproxylic species than high stumps. It can therefore be questioned whether high stumps are an optimal tool for nature conservation in clear-cutting forestry. Our results also indicate that low stumps constitute an equally important substrate as high stumps and logs, and we therefore suggest that stump harvesting is done after carefully evaluating measures to provide habitat for saproxylic organisms.

Introduction

Dead wood is a key factor in the preservation of biodiversity in forests [1–4]. In Fennoscandia, were forests are intensively managed, there has been a strong decline in the volumes of dead

wood [3,5,6], in particular the coarser fractions of dead wood in later stages of decay [6–9]. Biofuel harvesting has emerged in Swedish forestry as a possible way to help dampening the emissions of CO_2 [10], but at the same time it might pose further threat on forest biodiversity [11]. Currently, biofuels from Sweden's forests mainly come from logging residues on clear-cuts such as branches, twigs and tree tops (commonly referred to as "slash"). Low stumps are so far a minor, but rapidly growing biofuel source.

Dead wood on clear-cuts has qualities that are rare in mature forest; in particular warm and sun-exposed wood. Accordingly, the species compositions found in such substrates differ from those found in the cooler and moister forest interior [8,12–14]. Sun loving species that are favored or even dependent on large scale disturbances like fire might use clear-cuts as substitute [15], although the volumes of coarse woody debris (CWD) is substantially lower on clear-cuts. Up to 450 $m^3 ha^{-1}$ of CWD can be expected on fire fields previously dominated by high productive, old growth boreal spruce forest [3] compared to 11 $m^3 ha^{-1}$ found on boreal clear-cuts (low stumps excluded) [8]. Low stumps may constitute 80% of the CWD on slash harvested boreal clear-cuts [16,17] and 28% of the CWD on landscape level [11]. Snags, including high stumps, constitute a minute fraction of the dead wood on clear-cuts [8].

Species composition of saproxylic organisms may differ between low stumps and other substrate types on clear-cuts [18,19]. Some of the differences can probably be attributed to effects of diameter [19,20] while other might be due to microclimatic differences [21–25]. To gain better knowledge on the use of dead wood creation as a tool for nature conservation in clear-cutting forestry, saproxylic beetle communities in common dead wood substrates like low stumps, high stumps and logs should be carefully examined.

Hjältén et al. [18] investigated data from 2006 on assemblages of saproxylic beetles hatching from three different types of dead wood substrates (low stumps, high stumps and logs) on clear-cuts in the mid-boreal zone in Sweden. They found that species assemblages of saproxylic beetles differed significantly between substrate types. In the study presented here the same localities were re-sampled in 2007 and 2009. With this enlarged data set and a new analysis method we could conduct a repeated investigation of the species composition of these three substrate types, strengthening the inference on dead wood items on clear-cuts. We could also ensure that the differences found between substrate types in 2006 were not specific to, for example, the weather conditions in that particular year.

We asked the following questions: Firstly, are the differences in species assemblages between substrate types consistent over time. Secondly, do individual species or nutritional subgroups of saproxylic beetles show attraction to specific substrate types, e.g. are there low stump specialists? Lastly, we discuss the implications of the results for stump harvesting and forest management.

Materials and Methods

Ethics

The study was conducted on private land and permission for the study was given by the owners Sveaskog and Holmen Skog. In Sweden no ethical approval is needed for sampling beetles outside protected areas and the field study did not involve any endangered or protected species.

Study area and experimental design

The study was conducted at ten clear-cuts in the central-boreal zone [26] of Sweden, situated between 63°62'N and 64°29'N and 16°89'E and 20°13'E (Fig. 1). The altitude ranged from 100 to 550 m a.s.l. with an average of 337 m. The study is part of a large field experiment that was initiated in 2001 with experimentally introduced dead wood [8,14]. Prior to harvesting the stands were all dominated by Norway spruce (*Picea abies* (L.) Karst.). The surrounding forest

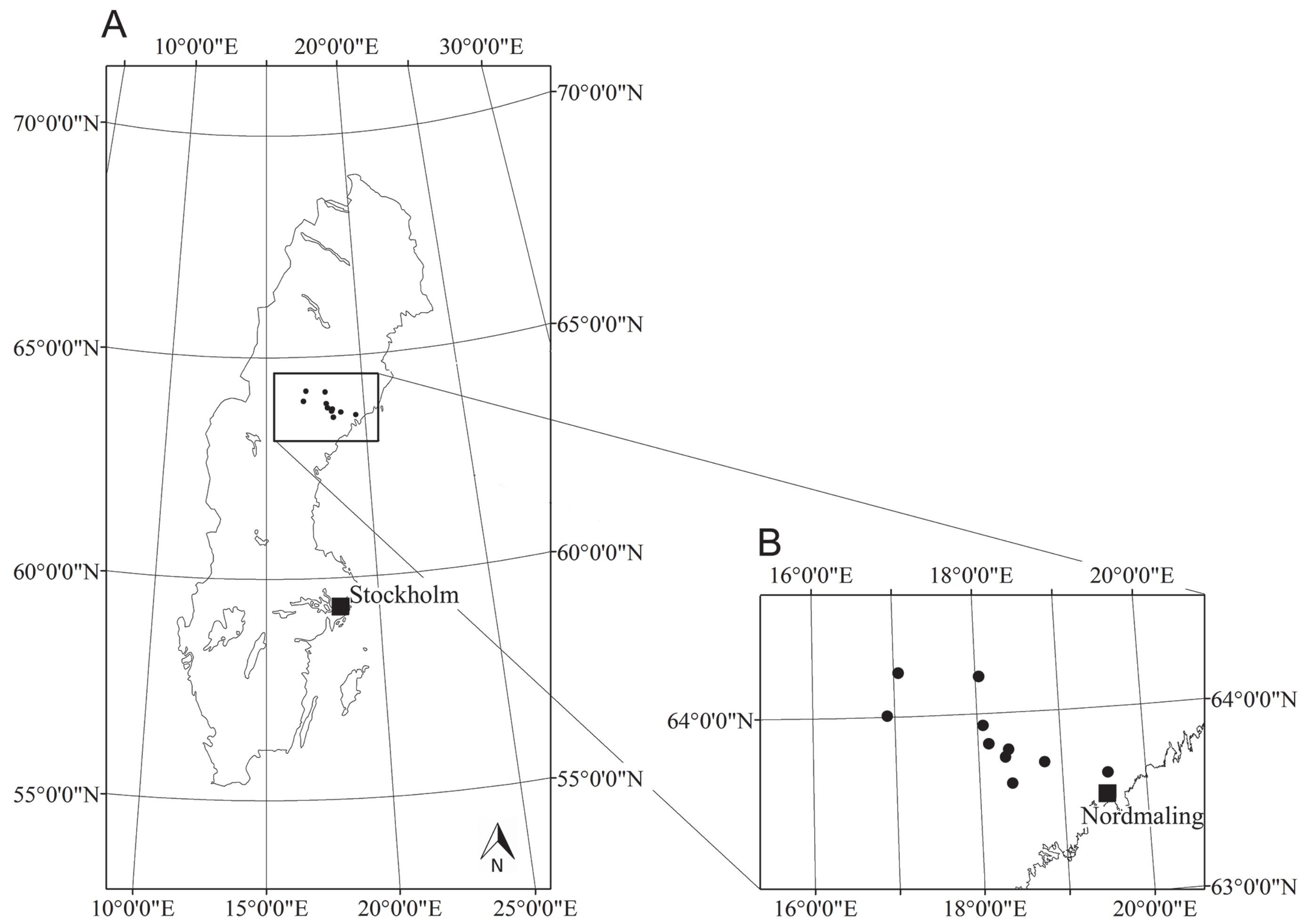

Fig 1. Study area. The location of the 10 clear-cuts in Sweden (A) and in the region (B). The distance between the vertical grid lines in B is approximately 50 km.

doi:10.1371/journal.pone.0118896.g001

mainly constituted of managed stands dominated by Norway spruce and Scots pine (*Pinus sylvestris* L.) in various age classes.

At each of the ten clear-cuts we created three high stumps of spruce (approximately 3 m in height and 21 cm in diameter at breast height) and we introduced three logs of spruce (4 m in length and approximately 21 cm in diameter). At each clear-cut we also used nine low stumps (three per sampling year) of spruce left after clear-cutting. These low stumps were located close to the other two substrate types. The low stumps and the high stumps were created at clear-cutting between 2000 and early 2001. All logs were introduced in early 2002 except in one clear-cut, where the logs were introduced in early 2001 [see 14 for details]. All ten clear-cuts were sampled in 2006, 2007 and 2009, approximately five to ten years after clear-cutting. Most of the substrates had some bark still remaining the last year of the study although in many cases detached from the wood. According to the description of successional phases of saproxylic beetles by Esseen et al. [27], our study would be placed in the transition between successional phase II and III. Phase II is characterized by species associated with early fungi growing under the loose bark, and secondary phloem-consuming species. These two groups do also have their own predators, different from the predators feeding on early phloem-consumers common in

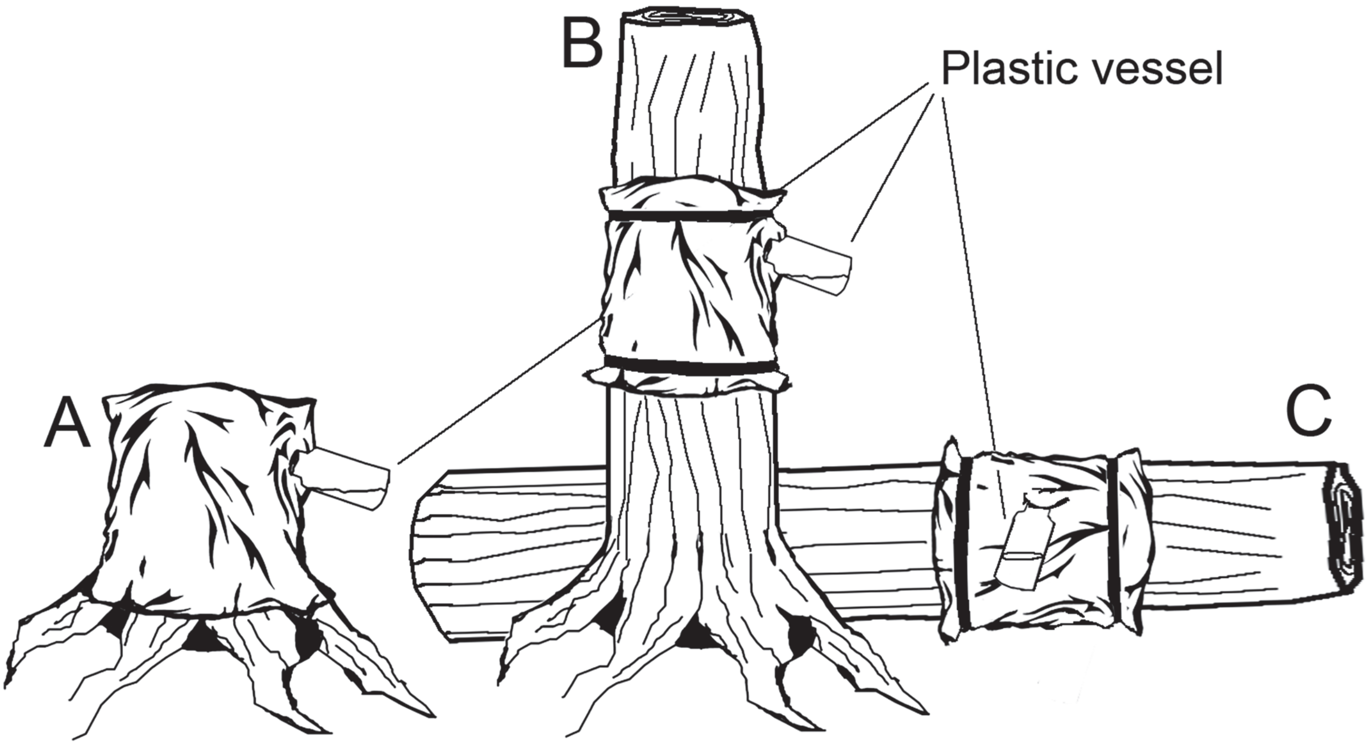

Fig 2. Substrate types and traps. Low stump (A), high stump (B) and log (C) with the emergence traps attached. Observe that the cut surface was covered by the trap only on low stumps. Note that in drawings B and C, the proportional length of the substrate covered by the trap is longer than in reality.

doi:10.1371/journal.pone.0118896.g002

successional phase I [28]. The subsequent, phase III coincides with a peak in the occurrence of wood fungi and is dominated by fungivores and their associated predators [27,28].

We sampled each substrate unit separately by using emergence traps. This trap type is useful when investigating substrate associations of saproxylic insects [29] because they almost exclusively trap individuals hatching from the sampled substrate. Furthermore, when the aim is to resample the same substrate several times the emergence trap is superior to e.g. sieving or debarking, which is likely to destroy sections of the substrate. The traps were made of black polypropylene weed barrier cloths which were wrapped around a defined section (approximately 20 cm for the low stumps, depending on height, and 30 cm for logs and high stumps) of each substrate type. The cut surface of the low stumps was covered by the trap (Fig. 2). At the top of each cloth we attached a white 250 ml plastic vessel filled to 1/3 with 50% propylene glycol and some detergent to break the surface tension. The diameter of the low stumps was often slightly wider than on logs and high stumps. On the other hand, low stumps were shorter than the enclosed parts of the logs and of the high stumps [see also 18]. To reduce effects of the trapping from previous years, we randomly moved the traps on logs and high stumps so that the same section was only used once. While moving the traps up and down the high stumps, no traps were put lower than 50 cm above ground and never higher than 50 cm below the top. For the same reason that we moved the traps on logs and high stumps, the same low stump was only sampled once during the three years. The sampling period ran from May to September in 2006, 2007 and 2009. The traps were emptied only once per year, in September.

Species determination and classification

The beetles were counted and identified to species level by experts. We defined saproxylic species according to Speight [30] and we used the saproxylic database [31,32] to get species specific information on connection to dead wood. The species were also classified into nutritional subgroups (S1 Table, including the list of references used to classify the species). The two dominating subgroups were: fungivores (mycetophagous species) and predators (predators and ectoparasitoids). Other nutritional subgroups, represented in smaller numbers, were cambivores (phloem and cortex feeders), detritrivores (necrophagous species) and wood borers (xylophagous species). The nutritional subgroups were partially overlapping because of multiple classifications for individual species (S1 Table). A small number of the species were not saproxylic and were neither included in the Supplementary material (S1 Table) nor in any analysis. Red-listed species were classified according to the 2010 Red List of Swedish Species [33]. Taxonomy and nomenclature of the beetles follows [34].

Statistical analysis

We used a general linear mixed effect model (GLMM) for poisson distributed response to analyze the effects of substrate type (high stumps, logs and low stumps) and year (2006, 2007 and 2009) and their possible interaction on the species richness of all saproxylic beetles, predatory, fungivorous and wood boring saproxylic beetles. Saproxylic cambivores and detritrivores were left out of this analysis because of few observations and a large number of zeroes. Instead of making a separate analysis on the relation between beetle abundance, years and substrate type, we made a univariate analysis on species level (see description below). Low stumps were in contrast to high stumps and logs not resampled. Instead new low stumps were sampled in 2007 and in 2009. Also, some traps were destroyed every year. After deleting data from destroyed and non-working traps (five high stumps, six logs and one low stump; two traps year 1, four traps year 2 and six traps year 4) we remained with three repeated measurements of 30 high stumps, 30 logs and 89 measurements of low stumps (see Table 1). Only in one case was the same substrate affected more than once (one log year 2 and year 4). In the GLMM analysis of species richness, locality and substrate identity were treated as random factors while substrate type and time were treated as fixed factors. Significant ($\alpha < 0.05$) results were examined with pair-wise Tukey tests. Mixed-effect models do, in contrast to standard repeated measures ANOVA, not require constant correlation structure between time points [35]. Thus, no test for sphericity was done. The residuals of each model were graphically studied and no obvious patterns could be seen.

To analyze effects from substrate type on community composition of saproxylic beetles we used multivariate generalized linear models (ManyGLM). By using corrections for poisson or negative binomial responses this relatively new method can handle zero inflated multiple

Table 1. The number of traps and substrates.

Substrate type	Yr1	Yr2	Yr4	# traps	# substrates
HS	30	28	27	85	30
L	28	28	28	84	30
LS	30	30	29	89	89
Total	88	86	84	258	149

In total we used 258 traps on 149 substrates on ten different clear-cuts. As each low stump was sampled only once, we sampled 90 different low stumps in total. The last year one trap on a low stump was destroyed.

doi:10.1371/journal.pone.0118896.t001

species count data, which often do not fulfill assumptions of multivariate normal distribution [36,37]. ManyGLM is also better than multivariate methods like PERMANOVA in detecting between group effects in less variable taxa [37]. However, as this method cannot yet handle mixed effect models we analyzed the effect of substrate type for each year separately. We created non-orthogonal contrasts to examine all pair-wise substrate combinations of significant ($\alpha < 0.05$) results in the main test. To analyze the effect of substrate type on individual species, the pair-wise comparisons were followed by the univariate test procedure implemented in ManyGLM. Because the number of comparisons was high and hence the detectability after adjustment for multiple comparisons very low we report unadjusted *p*-values for all tests, but interpret the results with caution [38–40]. To visualize the community composition in our substrate types we explored the data with non-metric multidimensional scaling (NMDS) [41]. We used the full assemblage in the NMDS ordination.

All statistical analyses were carried out in the open source software R [42]. The GLMMs were analyzed in the packages lme4 [43] and multcomp [44], the ManyGLM in the package mvabund [45] and the NMDS ordination was done in the package vegan [46].

Results

We trapped 2,670 saproxylic beetle individuals belonging to 195 species. Total saproxylic beetle abundance varied among substrate types (813 individuals in low stumps, 795 in high stumps and 1,062 in logs) and over years (872 in 2006, 1,105 in 2007 and 693 in 2009). Also, species richness varied among substrate types (62 species in low stumps, 72 in high stumps and 50 in logs). Among these specimens, 1,028 were classified as fungivores, 1,132 as predators, 615 as wood borers, 378 as cambivores and 24 as detritrivores. Seven species were not classified and for 25 species the classification was uncertain (S1 Table). High stumps produced 48 unique species (species only trapped on high stumps), while logs and low stumps produced 34 and 27 unique species respectively. The most abundant species was *Ampedus tristis* (15% of total abundance) followed by *Scaphisoma agaricinum* (9.4%) and *Crypturgus pusillus* (8.9%).

Abundance and species richness

Substrate type did not have a significant effect on total saproxylic species richness, but there were more saproxylic predators in high stumps and more saproxylic fungivores in logs and low stumps (Table 2). Sampling year had a significant effect on all groups except saproxylic fungivores and in all cases did the second sampling year stand out as a year with higher species richness (Table 2). No significant interaction between substrate type and year was found. We trapped 60 individuals of 20 nationally red-listed species; 10 species (17 individuals) from high stumps, 9 species (34 individuals) from logs, and 7 species (9 individuals) from low stumps. Four of these species (six individuals) are currently classified as vulnerable, 15 (53) as near threatened (NT) and one (one) as data deficient (DD) (S1 Table). The most common red-listed species was the predator *Lacon fasciatus* (NT) with 18 individuals, of which 11 hatched from logs (S1 Table). The number of red-listed species and individuals was, however, too low to allow for statistical analysis.

Species assemblages in different substrate types

The community composition of saproxylic beetles differed significantly between high stumps and logs all three years. However, the pair-wise comparisons showed that the species composition in low stumps was not different from high stumps, and different from logs only the second year (Table 3). The two-dimensional solution of the Bray-Curtis dissimilarity in the NMDS ordinations illustrates that the composition in stumps is intermediate to those in logs and high

Table 2. Species richness.

Source	Chi^2	P	post hoc test
**Time**			
All saproxylics	10.694	<**0.005**	Yr 2 > Yr 1, Yr 3
Predators	18.635	<**0.001**	Yr 2 > Yr 1, Yr 3
Fungivores	1.265	0.531	
Wood borers	20.084	<**0.001**	Yr 2 > Yr 1, Yr 3
**Substrate type**			
All saproxylics	1.282	0.527	
Predators	7.322	**0.026**	HS > L, LS
Fungivores	18.694	<**0.001**	L, LS > HS
Wood borers	3.217	0.200	

When significance (α < 0.05) occurred, we examined the effect of each factor level with Tukey tests. N = 10, i.e. each clear-cut was used as a replicate. HS > L denotes higher species richness in high stumps than in logs. HS = high stumps, L = logs and LS = low stumps.

The relation between substrate type, time, and the species richness of the three nutritional subgroups as described by the GLMM. For factors time and substrate, type *df* = 2 and for the interaction between time and substrate type *df* = 4. No significant result was encountered for this interaction and no result is reported.

doi:10.1371/journal.pone.0118896.t002

stumps (Fig. 3). Also, the NMDS plots indicate that assemblages in logs became more distinct in the two later years (Fig. 3).

There were 28 species that, according to the univariate analysis, responded significantly to the effect of substrate type one, at least one study year (Table 4). These species were in many cases also among the most common (S1 Table). Three of these species were caught all three years and all three were significantly more common on logs (Table 4). Five of the 28 species occurred all three years but was only significantly affected by substrate type two of these three years. Among these five species, three were significantly more common on one substrate type

Table 3. Species composition.

Source	df	wald	P	post hoc test
**Year 1**				
Substrate type	2	9.879	<**0.001**	HS ≠ L
Error	27			
**Year 2**				
Substrate type	2	10.25	<**0.001**	HS, LS ≠ L
Error	27			
**Year 3**				
Substrate type	2	8.645	<**0.003**	HS ≠ L
Error	27			

For the post hoc test the α-probability was set to 0.05 and N = 10, i.e. each clear-cut was used as a replicate. "≠"denotes significant difference between the substrates (abbreviations: HS = high stumps, L = logs and LS for = low stumps).

The ManyGLM analysis testing the relations between saproxylic beetle assemblages on clear-cuts and the studied substrate types.

doi:10.1371/journal.pone.0118896.t003

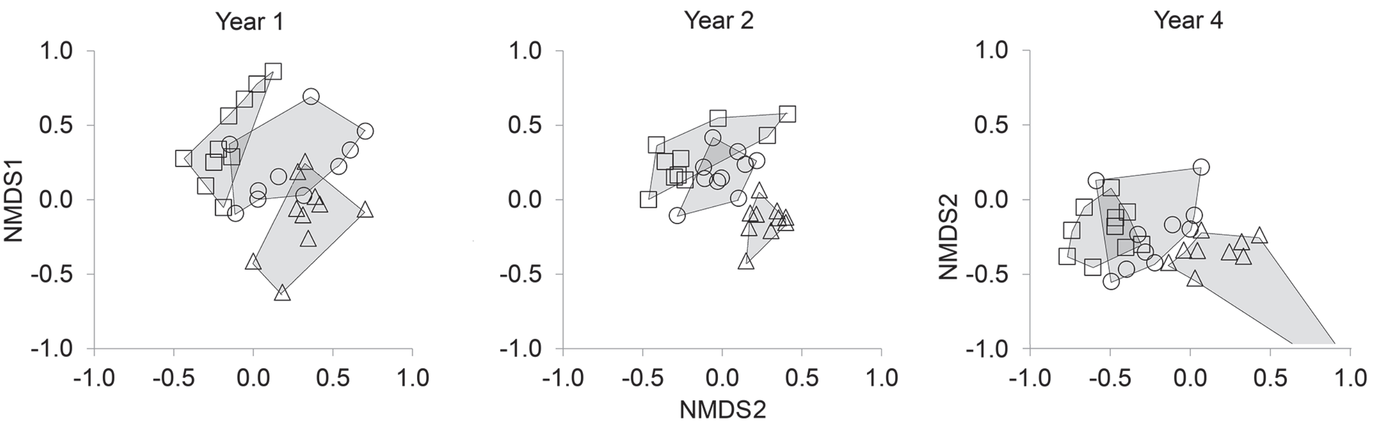

Fig 3. NMDS plots. The best two-dimensional NMDS solution of the difference in species assemblage. Each point represents one clear-cut and high stumps, logs and low stumps are denoted with squares, triangles, and circles respectively. Lines and shadows are drawn around each substrate type to the emphasize groupings. To be able to draw all plots on the same scale, one data point was left out in year. The 2D stress was 0.26.

doi:10.1371/journal.pone.0118896.g003

(one on each type), one was more common on logs and low stumps than on high stumps, and one showed inconsistent results. Two red listed species, *Corticaria interstitialis* (NT) and *Lacon fasciatus* (NT), were significantly more common on logs than on low stumps (Table 4). Among the remaining 18 species that were significantly affected by substrate type, four were more common on high stumps; six were more common on logs and seven more common on low stumps. The overall result from the analysis of individual species responses to substrate type suggests that high stumps were preferred by fewest species whereas logs were preferred by most. Low stumps were intermediate in this respect with fewer species preferences than logs but richer than high stumps.

Discussion

Nutritional subgroups

In consistence with previous results [47] we did not find significant differences between logs and low stumps when all species were included in the analysis. However, after dividing species into nutritional subgroups we found that fungivores and predators had different relationships to the studied substrate types. Fungivores were more species-rich on logs and low stumps and predators were more species-rich on high stumps. Taking one step down in the food chain could shed light on the observed pattern. Berglund et al. [48] found that although most of the investigated species of wood decaying fungi were not restricted to logs, they clearly favored this substrate type. It is possible that higher moisture and lower temperature in logs is responsible for this fungal pattern. Also, Jonsell and Weslien [22] (with support from studies by Thunes and Willassen [49] and Jonsell et al. [50]), suggested that the difference between substrate types they found in the abundance of single beetle species could be explained by differences in water content. In our study, both logs and low stumps were sampled close to the ground while high stumps were sampled higher up from the ground. As a consequence, low stumps and logs may have been more similar in terms of moisture level and supply of fungus, hence attracting more fungivorous beetles than high stumps. Yet, moisture level alone does not necessarily cause higher presence of saproxylic beetles [51]. Since the assemblage of beetles in high stumps is different at breast height than in the moister base [23], it is likely that the patterns in our study would have looked different if all high stumps had been sampled also close to the ground. The

Table 4. Individual species responses.

Species	Subgroup	Substrate	Year 1	Year 2	Year 4
Ampedus nigrinus	P, W	HS	ns	HS > L	HS > L
Anaspis rufilabris	P	HS	ns	-	HS > L, LS
Cis comptus	F	HS	ns	ns	HS > L, LS
Corticaria rubripes	F	HS	ns	HS > LS	ns
Nepachys cardiacae	P	HS	ns	HS > LS	ns
Atomaria bella	F	L	L > HS	L > HS	L > HS, LS
Curtimorda maculosa	F	L	L > HS	L > HS, LS	L > HS, LS
Ampedus tristis	P, W	L	L > HS	L > LS	ns
Anaspis bohemica	P	L	ns	L > HS, LS	ns
Tyrus mucronatus	?F	L	ns	L > HS	ns
Crypturgus pusillus	C	L	L > LS	ns	ns
Dryocoetes autographus	C	L	L > HS	ns	ns
Atomaria subangulata	F	L	ns	L > HS	ns
Cis boleti	F	L	L > LS	ns	ns
Corticaria interstitialis	F	L	ns	L > LS	ns
Gyrophaena strictula	F	L	L > HS	ns	-
Lacon fasciatus	P	L	ns	L > LS	-
Asemum striatum	C, W	L, LS	L > LS > HS	LS > L	ns
Scaphisoma agaricinum	F	L, LS	L, LS > HS	L, LS > HS	L, LS > HS
Anisotoma axillaris	F	LS	ns	LS > HS	LS > HS
Cerylon histeroides	F	LS	ns	ns	LS > HS, L
Enicmus rugosus	F	LS	LS > L	ns	ns
Sphindus dubius	F	LS	ns	LS > L	ns
Atheta sg. Alaobia sodalis	F,? D, P	LS	-	ns	LS > HS
Bibloporus bicolor	P	LS	ns	LS > L	ns
Euplectus punctatus	P	LS	ns	LS > L	ns
Melanotus castanipes	P, W	LS	ns	LS > L	ns
Anaspis marginicollis	P	i	L, LS > HS	LS > HS > L	ns

Abbreviations for nutritional subgroups: C = cambivore, D = detritrivore, F = fungivore, P = predator, and W = wood borer. "?" before a classification means that the classification is uncertain; and for substrate types: HS = high stumps, L = logs and LS for = low stumps. HS > L denotes higher abundance in high stumps than in logs. "i" and "ns" denotes inconclusive result and non-significant result respectively and "-"indicate absence of the species.

Species that, according to the univariate pair-wise tests, significantly ($\alpha < 0.05$) responded to the different substrate types ordered from top to bottom in respect to their respective substrate preference and according to the number of years the response was significant (all years, two years or one year).

doi:10.1371/journal.pone.0118896.t004

predator group were in contrast to fungivores more species rich on high stumps. We have no good explanation for this result.

Beetle assemblages and substrate types

Our results, that low stumps, snags and logs on clear-cuts are inhabited by differing assemblages of saproxylic beetles, conforms with the results from previous studies [18,23,47]. Adding the data from 2007 and 2009 to the 2006 data already published in Hjältén et al. [18] revealed (i) that assemblages in low stumps are similar to those in high stumps (Fig. 3, Table 3), (ii) that the species assemblages of logs is more distinct in relation to the ones found in high and low stumps, and (iii) that individual species responses to substrate type are consistent over time

(Table 4). In earlier comparisons of high stumps and low stumps, results have shown that species assemblages, although widely overlapping, differ significantly in these two substrate types. Our new insights, in combination with the previous ones, lead us to the suggestion that high stumps and low stumps may lose their specific features with time. The result in the first year, which was also analyzed in Hjältén et al. [18], was different in this new study; no significant difference between high and low stumps. The divergence is hard to explain, but we used a different analysis which might be more sensitive than the PERMANOVA used in Hjältén et al. [18]. After comparing the ManyGLM result with the NMDS plots (Table 3, Fig. 3) it is clear that the results are coinciding; that assemblages in low stumps and high stumps are more alike and that assemblages in logs are more distinct. Also, our new result is similar all three years which further suggests that this pattern is correct.

Responses by individual species

The repeated inference of substrate preference, as the one made for *A. bella*, *C. maculosa* and *S. agaricinum* (significantly affected all three years) and *A. nigrinus*, *A. tristis*, *A. axillaris* and *A. striatum* (significantly affected two of three years) suggests that substrate preferences change little over time. If this is true for other less common species remains unknown. As with the nutritional subgroups it might be useful to look for answers elsewhere in the food chain. For example, the obligate saproxylic fungivore, *Curtimorda maculosa* was one of the two species that were significantly more common on logs all three years. The species was during the same period never found on high stumps. According to Dahlberg and Stokland [32] *C. maculosa* is strictly associated with the bracket fungi *Gloeophyllum sepiarium* which is commonly found on dry, sun exposed dead wood on clear-cuts. Furthermore, this bracket fungi is found more often on logs than on low stumps and standing dead wood [52]. Another example of a species found significantly more often on logs was the fungivore *S. agaricinum*. In an earlier study conducted with same methods and on the same clear-cuts as the ones in the study presented here, it was found that this species favored logs before high stumps [14].

Eight species were significantly more common on low stumps. The most common of these was the saproxylic fungivore *E. rugosus*. This species have been found to favor top boles of spruce before high stumps in one year old substrates [53], high stumps at ground level before low stumps in one to three year old substrates [23] and low stumps before logs in four to five year old substrates [47]. The result from studying our four to nine year old substrates strongly agrees with that of Jonsell and Hansson [47] that low stumps is the favored substrate type in intermediate stages of decay. *Anisotoma axillaris*, was significantly more common on low stumps than on high stumps two of these three years. Here we can make an interesting comparison between our data on *A. axillaris* and results published on a large data set from the same area: In Hjältén et al. [14] logs and high stumps with a variety of features were placed in three different forest types and sampled four different years over a period of six years (2001 to 2006). They caught 20 individuals of *A. axillaris* on logs and high stumps. In the study presented here, 59 individuals of this species were caught; all but five on low stumps (S1 Table). Hence, low stumps appear to be a very important substrate for this species. *Enicmus rugosus*, on the other hand, is very common and also reproduce on other substrate types than low stumps, it is unlikely that this species would perish if stump harvesting becomes widespread.

The species *Ampedus nigrinus*, *Anaspis rufilabris*, *Cis comptus*, *Corticaria rubripes* and *Nepachys cardiace* were all significantly more common on high stumps (Table 4). Fäldt et al. [54] evaluated the attraction of different beetle species to the chemical compounds found in the volatiles released from two commonly occurring species of bracket fungi; *Fomes fomentarius* and *Fomitopsis pinicola*. *A. rufilabris* was significantly attracted to the chemical compounds

released from *F. pinicola*. The bracket fungi *F. pinicola* is very common brown rotting species which is abundantly occurring on high stumps as well as other substrate types on clear-cuts [22,48,55,56] and a possible positive correlation between stump height and this species have been suggested [56]. This species may therefore be more common on high sumps than on logs. However, we did not investigate the occurrence or the possible substrate preference of any bracket fungi; hence we cannot tell if the substrate preference by *A. rufilabris* was due to higher occurrence of *F. pinicola* on our high stumps. The larva of the very common Elaterid species *A. nigrinus* is a predator living in brown rotted wood while feeding on the larvae of other saproxylic insect species. Also in this case, *F. pinicola* may have caused the pattern. Yet another species which have been shown to favor high stumps is the fungivore *Hadreule elongatula* [57]. We only caught three individuals of this species, all on high stumps.

Low stumps are difficult to sample due to the complex structures in this part of the tree. For the same reason, it is also difficult to ensure that the same mantle area or volume is sampled in low stumps compared to the more simply shaped high stumps and logs. Therefore, the comparison between stumps and other substrates is not a straight-forward one, neither in the study presented here nor in previous studies. We judge, however, that at least the results concerning species composition mirrors the real differences between the aboveground parts of low stumps, high stumps and logs.

Implications for sustainable forest management

Our study shows that, in varying degree, beetle assemblages differ between different substrate types. This implies that some species cannot use one substrate type as a substitute for another; a fact which has been carefully noted in previous studies [53,58,59]. We suggest that forest management should create a variety of different substrate types, more than is done today. High stumps of conifers, currently common in the present forestry in this region, are important, but according to our results they should be complemented with logs (see also [58]) and dead wood from other tree species or with varying diameters [20,60]. Low stumps represent a large proportion of the coarse dead wood found on clear-cuts [19,61,62] and they might constitute the dominating substrate for saproxylic species associated with dry and warm dead wood on landscape level. Red listed species does also reproduce in low stumps. In our study, nine beetle individuals of eight red-listed species hatched from 89 low stumps during three growing seasons (S1 Table), which might sound like a minor issue. But it translates into one red-listed beetle per ten low stumps and potentially thousands of red listed beetles on large clear-cuts. In areas with very low amounts of coarse dead wood like the forests in Sweden, clear-cuts may be the last outpost for some warmth demanding saproxylic species. We therefore recommend that stump harvesting should be used with absolute caution until a more thorough evaluation of the importance of low stumps for red listed species is done. We also recommend that high stumps are complemented with logs.

Supporting Information

S1 Table. The frequency distribution, taxonomic family, saproxylic group, nutritional ecology and red list class of the all the saproxylic beetles caught during the three sampling years 2006, 2007 and 2009. Species are ordered taxonomically according to *Catalogus Coleopterorum Sueciae*. Abbreviations for 1. nutritional ecology: D = detritus feeder; F = fungivore; H = herbivore; P = predator; W = wood-borer (for references see details under the species list), 2. saproxylic group: SxF = facultatively saproxylic; SxO = obligate saproxylic is according to the saproxylic data base (http://www.saproxylic.org) and 3. red list class: DD = data deficient; NT = nearly threatened; VU = vulnerable is are according to Gärdenfors (2010).
(XLSX)

Acknowledgments

The study was kindly supported by The Swedish Energy Agency. We would also like to thank the forestry companies Sveaskog and Holmen for letting us use their land in this study. Big thanks to Stig Lundberg and Jacek Hilszczański for identification of all beetle specimens. We also thank Åke Nordström and Therese Johansson for helping us with the field work.

Author Contributions

Conceived and designed the experiments: JH. Performed the experiments: JA JH. Analyzed the data: JA. Contributed reagents/materials/analysis tools: JH JA MD. Wrote the paper: JA JH MD.

References

1. Berg A, Ehnström B, Gustafsson L, Hallingbäck T, Jonsell M, Weslien J. Threatened plant, animal, and fungus species in swedish forests—Distribution and habitat associations. Conserv Biol. 1994; 8: 718–731.
2. Esseen P-A, Ehnström B, Ericson L, Sjöberg K. Boreal forests. Ecol Bull. 1997; 46: 16–47.
3. Siitonen J. Forest management, coarse woody debris and saproxylic organisms: Fennoscandian boreal forests as an example. Ecol Bull. 2001; 49: 11–41.
4. Grove SJ. Saproxylic insect ecology and the sustainable management of forests. Annu Rev Ecol Syst. 2002; 33: 1–23.
5. Linder P, Elfving B, Zackrisson O. Stand structure and successional trends in virgin boreal forest reserves in Sweden. Forest Ecol Manag. 1997; 98: 17–33.
6. Linder P, Östlund L. Structural changes in three mid-boreal Swedish forest landscapes, 1885–1996. Biol Conserv. 1998; 85: 9–19.
7. Kruys N, Jonsson BG. Fine woody debris is important for species richness on logs in managed boreal spruce forests of northern Sweden. Can J Forest Res. 1999; 29: 1295–1299.
8. Stenbacka F, Hjältén J, Hilszczański J, Dynesius M. Saproxylic and non-saproxylic beetle assemblages in boreal spruce forests of different age and forestry intensity. Ecol Appl. 2010; 20: 2310–2321. PMID: 21265460
9. Ekbom B, Schroeder LM, Larsson S. Stand specific occurrence of coarse woody debris in a managed boreal forest landscape in central Sweden. Forest Ecol Manag. 2006; 221: 2–12.
10. Walmsley JD, Godbold DL. Stump Harvesting for Bioenergy—A Review of the Environmental Impacts. Forestry. 2010; 83: 17–38.
11. Bouget C, Lassauce A, Jonsell M. Effects of fuelwood harvesting on biodiversity—a review focused on thesituation in Europe. Can J Forest Res. 2012; 42: 1421–1432.
12. Johansson T, Hjältén J, Hilszczański J, Stenlid J, Ball JP, Alinvi O, et al. Variable response of different functional groups of saproxylic beetles to substrate manipulation and forest management: Implications for conservation strategies. Forest Ecol Manag. 2007; 242: 496–510.
13. Kaila L, Martikainen P, Punttila P. Dead trees left in clear-cuts benefit saproxylic Coleoptera adapted to natural disturbances in boreal forest. Biodivers Conserv. 1997; 6: 1–18.
14. Hjältén J, Stenbacka F, Pettersson R, Gibb H, Johansson T, Danell K, et al. Micro and Macro-Habitat Associations in Saproxylic Beetles: Implications for Biodiversity Management. PLoS ONE. 2012; 7: e41100. doi: 10.1371/journal.pone.0041100 PMID: 22848432
15. Gibb H, Pettersson RB, Hjältén J, Hilszczański J, Ball JP, Johansson T, et al. Conservation-oriented forestry and early successional saproxylic beetles: Responses of functional groups to manipulated dead wood substrates. Biol Conserv. 2006; 129: 437–450.
16. Dahlberg A, Thor G, Allmer J, Jonsell M, Jonsson M, Ranius T. Modelled impact of Norway spruce logging residue extraction on biodiversity in Sweden. Can J Forest Res. 2011; 41: 1220–1232.
17. Geijer E, Andersson J, Bostedt G, Brännlund R, Hjältén J. Safeguarding Species Richness vs. Increasing the use of Renewable Energy—the Effect of Stump Harvesting on Two Environmental Goals. J Forest Econ. 2014; 20: 111–125.
18. Hjältén J, Stenbacka F, Andersson J. Saproxylic beetle assemblages on low stumps, high stumps and logs: Implications for environmental effects of stump harvesting. Forest Ecol Manag. 2010; 260: 1149–1155.

19. Caruso A, Rudolphi J, Thor G. Lichen species diversity and substrate amounts in young planted boreal forests: A comparison between slash and stumps of *Picea abies*. Biol Conserv. 2008; 141: 47–55.
20. Jonsell M, Hansson J, Wedmo L. Diversity of saproxylic beetle species in logging residues in Sweden—Comparisons between tree species and diameters. Biol Conserv. 2007; 138: 89–99.
21. Sverdrup-Thygeson A, Ims RA. The effect of forest clearcutting in Norway on the community of saproxylic beetles on aspen. Biol Conserv. 2002; 106: 347–357.
22. Jonsell M, Weslien J. Felled or standing retained wood—it makes a difference for saproxylic beetles. Forest Ecol Manag. 2003; 175: 425–435.
23. Abrahamsson M, Lindbladh M. A comparison of saproxylic beetle occurrence between man-made high- and low-stumps of spruce (Picea abies). Forest Ecol Manag. 2006; 226: 230–237.
24. Russo D, Cistrone L, Garonna AP. Habitat selection in the highly endangered beetle Rosalia alpina: a multiple spatial scale assessment. J Insect Conserv. 2011; 15: 685–693.
25. Bosso L. Rebelo H. Garonna AP. Russo D. Modelling geographic distribution and detecting conservation gaps in Italy for the threatened beetle Rosalia alpina. J Nat Conserv. 2013; 21:72–80.
26. Ahti T, Hämet-Ahti L, Jalas J. Vegetation zones and their sections in northwestern Europe. Ann Bot Fenn. 1968; 3: 169–211.
27. Esseen PA, Ehnström B, Ericson L, Sjöberg K. Boreal forests—the focal habitats of Fennoscandia. In: Hansson L, editors. Ecological Principles of Nature Conservation. Springer US, 1992. pp. 252–325.
28. Stokland JN, Siitonen J. Mortality factors and decay succession. In: Stokland JN, Siitonen J, Jonsson BG, editors. Biodiversity in Dead Wood. New York: Cambridge University Press. 2012. pp. 110–149.
29. Wikars LO, Sahlin E, Ranius T. A comparison of three methods to estimate species richness of saproxylic beetles (Coleoptera) in logs and high stumps of Norway spruce. Can Entomol. 2005; 137: 304–324.
30. Speight MCD. Saproxylic Invertebrates and their Conservation. Strasbourg. Council of Europe. 1989.
31. Anon. The Saproxylic Database—Biodiversity in Dead Wood; 2007. Database: [Internet]. Accessed: http://radon.uio.no/WDD/Login.aspx?ReturnUrl=%2Fwdd%2FDefault.aspx.
32. Dahlberg A, Stokland J. Vedlevande arters krav på substrat—Sammanställning och analys av 3 600 arter. Jönköping. Skogsstyrelsen. 2004. [In Swedish]
33. Gärdenfors U. The 2010 red list of Swedish species. Uppsala. Artdatabanken. 2010.
34. Silfverberg H. Enumeratio nova Coelopterorum Fennoscandiae, Daniae et Baltiae. Helsingfors. 2004.
35. Lindström ML, Bates DM. Nonlinear mixed effects models for repeated measures data. Biometrics. 1990; 46: 673–687. PMID: 2242409
36. Wang Y, Naumann U, Wright ST, Warton DI. mvabund—an R package for model-based analysis of multivariate abundance data. Methods Ecol Evol. 2012; 3: 471–474.
37. Warton DI, Wright ST, Wang Y. Distance-based multivariate analyses confound location and dispersion effects. Methods Ecol Evol. 2012; 3: 89–101.
38. Garcia LV. Escaping the Bonferonni iron claw in ecological studies. Oikos. 2004; 105: 657–663.
39. Perneger TV. What's wrong with Bonferroni adjustments. Brit Med J. 1998; 316: 1236–1238. PMID: 9553006
40. Nakagawa S. A farewell to Bonferroni: the problems of low statistical power and publication bias. Behav Ecol. 2004; 15: 1044–1045.
41. Kruskal JB. Nonmetric multidimensional-scaling—a numerical-method. Psychometrika. 1964; 29, 115–129.
42. R Development Core Team. R: a language and environmental for statistical computing.
43. Bates D, Maechler M, Bolker B, Walker S. lme4: Linear mixed-effects models using Eigen and S4. package version 1.0–4.
44. Hothorn T, Bretz F, Westfall P. Simultaneous Inference in General Parametric Models. Biom J. 2008; 50: 346–363. doi: 10.1002/bimj.200810425 PMID: 18481363
45. Wang Y, Naumann U, Wright S, Warton D. mvabund: statistical methods for analysing multivariate abundance data. R package version 3.8.3.
46. Oksanen J, Blanchet FG, Kindt R, Legendre P, Minchin PR, O'Hara, RB, et al. vegan: Community Ecology Package. R package version 2.0–9.
47. Jonsell M, Hansson J. Logs and Stumps in Clearcuts Support Similar Saproxylic Beetle Diversity: Implications for Bioenergy Harvest. Silva fenn. 2011; 45: 1053–1064.

48. Berglund H, Jonsson M, Penttilä R, Vanha Majamaa I. The effects of burning and dead-wood creation on the diversity of pioneer wood-inhabiting fungi in managed boreal spruce forests. Forest Ecol Manag. 2011; 261: 1293–1305.

49. Thunes KH, Willassen E. Species composition of beetles (Coleoptera) in the bracket fungi Piptoporus betulinus and Fomes fomentarius (Aphyllophorales: Polyporaceae): An explorative approach with canonical correspondence analysis. J Nat Hist. 1997; 31: 471–486.

50. Jonsell M, Nordlander G, Ehnström B. Substrate associations of insects breeding in fruiting bodies of wood-decaying fungi. Ecol Bull. 2001; 49: 173–194.

51. Ols C, Victorsson J, Jonsell M. Saproxylic insect fauna in stumps on wet and dry soil: Implications for stump harvest. Forest Ecol Manag. 2013; 290: 15–21.

52. Berglund H, Hottola J, Penttilä R, Siitonen J. Linking substrate and habitat requirements of wood-inhabiting fungi to their regional extinction vulnerability. Ecography. 2011; 34: 864–875.

53. Fossestøl K, Sverdrup-Thygeson A. Saproxylic beetles in high stumps and residual downed wood on clear-cuts and in forest edges. Scandinavian J For Res. 2009; 24: 403–416.

54. Fäldt J, Jonsell M, Nordlander G, Borg-Karlson AK. Volatiles of bracket fungi Fomitopsis pinicola and Fomes fomentarius and their functions as insect attractants. J Chem Ecol. 1999; 25: 567–590.

55. Djupström LB, Weslien J, Hoopen JT, Schroeder LM, ten Hoopen J. Restoration of habitats for a threatened saproxylic beetle species in a boreal landscape by retaining dead wood on clear-cuts. Biol Conserv. 2012; 155: 44–49.

56. Weslien J, Djupström LB, Schroeder M, Widenfalk O. Long-term priority effects among insects and fungi colonizing decaying wood. J Anim Ecol. 2011; 80: 1155–1162. doi: 10.1111/j.1365-2656.2011.01860.x PMID: 21569031

57. Schroeder L, Ranius T, Ekbom B, Larsson S. Recruitment of saproxylic beetles in high stumps created for maintaining biodiversity in a boreal forest landscape. Can J Forest Res. 2006; 36: 2168–2178.

58. Jonsson BG, Kruys N, Ranius T. Ecology of species living on dead wood—Lessons for dead wood management. Silva fenn. 2005; 39: 289–309.

59. Franc N. Standing or downed dead trees—does it matter for saproxylic beetles in temperate oak-rich forest? Can J Forest Res. 2007; 37: 2494–2507.

60. Lindhe A, Lindelöw A. Cut high stumps of spruce, birch, aspen and oak as breeding substrates for saproxylic beetles. Forest Ecol Manag. 2004; 203: 1–20.

61. Egnell G, Hyvönen R, Högbom L, Johansson T, Lundmark T, Olsson B, et al. Environmental aspects on stump-harvest—compilation of knowledge and knowledge gaps. Energimyndigheten. 2007:40. Available: http://www.slu.se/PageFiles/269289/ER%202007_40W.pdf. [In Swedish]

62. Eräjää S, Halme P, Kotiaho J, Markkanen A, Toivanen T. The Volume and Composition of Dead Wood on Traditional and Forest Fuel Harvested Clear-Cuts. Silva fenn. 2010; 44: 203–211.
